# The role of glycans in personalization of preventive health care

**DOI:** 10.3325/cmj.2024.65.293

**Published:** 2024-06

**Authors:** Gordan Lauc, Dragan Primorac

**Affiliations:** 1 Faculty of Pharmacy and Biochemistry, University of Zagreb, Zagreb, Croatia *glauc@pharma.hr*; 2 St. Catherine Specialty Hospital, Zabok/Zagreb, Croatia; 3 Eberly College of Science, The Pennsylvania State University, University Park, PA, USA; 4 University of Split, School of Medicine, Split, Croatia; 5 University of Osijek, School of Medicine, Osijek, Croatia

More than 20 years have passed since the late Dr Allen Roses, then the worldwide vice-president of genetics at GlaxoSmithKline, sent shockwaves through the medical community by openly stating that “the vast majority of drugs—more than 90 percent—only work in 30 to 50 percent of people.” Since then, this has become widely accepted as fact. Today, companion diagnostic procedures exist for many drugs, and pharmacogenomics is becoming the standard of care in numerous hospitals (1,2). However, while curative medicine progressively embraces an individualized approach, preventive strategies are lagging.

Nearly all existing health care systems operate on the premise of treating individuals after they fall ill, relying on periodic interventions when health deteriorates and impedes normal functioning. However, this approach is becoming increasingly financially unsustainable, and global health care funding remains in a perpetual state of crisis. The EU4Health program, established by Regulation (EU) 2021/522 on March 24, 2021, emphasizes the significance of shifting the focus toward disease prevention and health promotion. This shift entails continuous preventive health care tailored to individual needs.

There is no doubt that people are different and that interindividual differences strongly affect our metabolism. For example, the metabolic response to the same food may vary significantly even between identical twins, which indicates that interindividual differences surpass genetic differences (3,4). However, epidemiological studies are generally not stratified based on any biomarker, so personalization of the effects of dietary and other lifestyle interventions on health requires additional research.

Variation in our genes is important for the personalization of lifestyle interventions, but it only captures one aspect of disease risk (2). The influence of lifestyle on the majority of complex diseases is profound, with evidence indicating that a person’s lifespan could be extended by up to a decade or more through adherence to healthy dietary patterns (5). The elucidation of molecular mechanisms responsible for these effects has commenced (6). Since our genome does not change with time, the effects of lifestyle on disease risk cannot be estimated by analyzing genetic variants. The study subjects of three types of omics: glycome (complex oligosaccharides that are covalently attached to proteins), metabolome (set of small molecules that can be quantified by nuclear magnetic resonance or mass spectrometry), and gut metagenome (genome of all microorganisms living in our intestines), are possible candidates for quantification of disease risk in a dynamic manner. Among them, glycome is the most promising since glycans modulate the function of most proteins and are produced in a complex process affected by genetic, epigenetic, and environmental factors (7), while at the same time they are chemical structures that are very stable and can be reliably quantified.

Glycosylation is the most elaborate posttranslational modification of proteins that significantly affects their structure and function. Immunoglobulin G (IgG) is the most abundant antibody, representing 75% of all immunoglobulins and 10%-20% of total protein. Each IgG heavy chain contains a conserved *N*-glycosylation site on Asn-297. By altering protein structure, these glycans change binding affinity to different IgG receptors, which affects nearly all biological processes that involve IgG (8). IgG glycome is characterized by significant inter-individual variability, with galactosylation being the most variable feature. The composition of the IgG glycome gradually changes with age and the progression of certain inflammatory conditions. However, some strong immune and physiological triggers, such as cardiac or bariatric surgery, severe infection, or sex hormone interventions, can result in rapid and extensive changes (9). The composition of the glycome is influenced by genes and the environment through epigenetic mechanisms. Genome-wide association studies have revealed that IgG glycosylation is regulated by a large network of genes with extensive pleiotropy with a number of age- and inflammation-related diseases (10). IgG glycosylation modulates effector functions of IgG, which makes glycans functional effectors that regulate inflammation at multiple levels. For example, galactosylation affects IgG inflammatory potential by modulating binding affinities to downstream effector molecules, including components of complement Fc receptors. Another IgG glycan modification, the addition of sialic acid, influences the capacity of the Fc domain to alternate between conformational states and may act as a switch between receptor specificities, resulting in opposing immunological outcomes. Therefore, glycosylation of IgG is not only an excellent biomarker of inflammation, but also a functional effector associated with aging and environmental influences on the transition of health to disease state. Glycosylation of many other proteins is also relevant as a functional effector and a biomarker of different diseases (7).

An age-related gradual decrease in galactosylated IgG, and the resulting build-up of agalactosylated IgG, is not only a hallmark of chronic systemic inflammation but it also exacerbates inflammation. In a vicious self-fueling loop, agalactosylated IgG acts as both a biomarker of aging and a pro-inflammatory effector. Low-level inflammation has indeed been proven to be a good predictor of healthy aging, which confirms the association between systemic chronic inflammation and the risk of age-related diseases (11). The first study reporting IgG glycosylation changes in disease was published in 1985, when it was discovered that IgG glycans are altered in rheumatoid arthritis and osteoarthritis (12). Since then, changes in glycosylation have been reported in most diseases that were investigated (9).

In many diseases, changes in IgG glycosylation resemble accelerated aging, which likely reflects increased chronic systemic inflammation contributing to disease development. Consequently, many diseases are associated with similar changes in the IgG glycome, which limits the use of the IgG glycome as a specific diagnostic biomarker for any of these diseases. However, since changes in IgG glycosylation significantly affect the immune system in general, quantifying IgG glycome composition enables the stratification of patients based on the inflammatory potential of their IgG. In many diseases, glycosylation changes at least five to ten years before the disease is diagnosed, which provides an early warning signal and opens a possibility of preventive interventions (9).

One of the most promising short-term steps that may advance personalized preventive medicine is the development and implementation of predictive biomarkers (13), where glycans have the highest diagnostic potential. Identifying individual variations in glycosylation patterns facilitates the optimization of preventive strategies based on an individual's distinct molecular profile. Such personalized approaches could identify at-risk individuals earlier, optimize preventive measures, and enhance the efficacy of individualized health care strategies.

**Figure 1:**
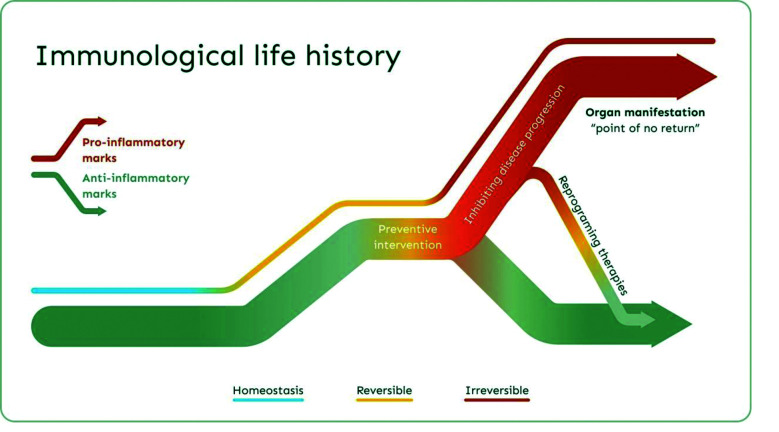
Chronic systemic inflammation is underlying most noncommunicable diseases that burden the aging population. Most often it is addressed only after it passes through the “point of no return” and manifests in a form of some common chronic diseases. However, if biomarkers indicative of systemic chronic inflammation would be timely monitored, then simple lifestyle of preventive pharmacological interventions could reverse this trend and prevent, or at last postpone, the onset of chronic diseases.

Increasing age and unhealthy lifestyles are associated with both higher circulating levels of inflammatory biomarkers (inflammaging) and a higher incidence of multiple noncommunicable diseases that have chronic systemic inflammation as a part of their molecular pathophysiology (Figure 1). However, in most cases, the causes of increase in different biomarkers are not clear, and it is unknown whether interventions improving these biomarkers would also decrease the disease risk or severity. For example, dietary supplements that affect DNA methylation can change the epigenetic clock of aging (14), but it remains unclear whether this might have any health benefits. Glycans are here an exception, since specific glycosylation patterns on circulating plasma proteins (including IgG) are altering functional properties of these proteins and thus may serve as early indicators and a dynamic measure of increased risk for cardiovascular disease or type 2 diabetes, guiding early preventive measures (15,16). For example, animal studies indicated that dietary supplementation with *N*-acetylmannosamine and consequential improvement in IgG sialylation can prevent the development of hypertension in obese mice (17). The same effect was observed for prevention of insulin resistance (18). Furthermore, in autoimmune disorders such as rheumatoid arthritis, inflammatory bowel disease or type 1 diabetes, specific glycosylation changes on antibodies contribute to disease onset and progression, offering potential targets for personalized therapeutic interventions (19-22). Additionally, as glycosylation is responsive to various stimuli, its changes could be used to monitor the impact of distinct environmental exposures. For instance, certain lifestyle interventions, including caloric restriction, physical exercise and weight loss, induced favorable changes in glycosylation of circulatory glycoproteins (3). Hence, mapping the dynamics of glycan patterns would allow for tailored interventions, such as personalized dietary recommendations, optimized preventive strategies, or targeted drug therapies.

Glycans are the leading class of molecules for monitoring the impact of lifestyle interventions at the individual level. GlycanAge test of biological age is today available in over 1000 clinics all around the world. At the moment, glycan analysis is not a diagnostic test, but the IgG glycome composition obtained as a measure of biological age can be interpreted by a licensed physician in the context of published scientific literature and be used to provide personalized advice. The importance of glycans in monitoring aging at the individual level and proactive prevention of age-related diseases was clearly identified in the recent perspective paper in The Cell by the Consortium for Biomarkers of Aging, which stated that GlycanAge is the only biomarker of age that correlates with numerous diseases and that is being commercially used to monitor lifestyle interventions (23). Recent Technology Feature “Aging research comes of age” published in Nature Methods also featured glycans as a tool to quantify aging process and monitor systemic chronic inflammation that develops with age (24). The translation of glycan biomarkers from a research tool into a routine clinical diagnostic is expected to enable significant progress in the personalization of different preventive lifestyles and pharmacological interventions.
